# Reliability and validity of the Chinese version of a short form of the family health scale

**DOI:** 10.1186/s12875-022-01702-1

**Published:** 2022-05-06

**Authors:** Fei Wang, Yunchou Wu, Xiaonan Sun, Dong Wang, Wai-Kit Ming, Xinying Sun, Yibo Wu

**Affiliations:** 1 State Key Laboratory of Cognitive Neuroscience and Learning, Beijing Normal University, Beijing, China; 2 School of Philosophy, Anhui University, Hefei, China; 3 School of Humanities and Social Sciences, Harbin Medical University, Harbin, China; 4 School of Public Health, Shaanxi University of Chinese Medicine, Xianyang, China; 5 Department of Infectious Diseases and Public Health, Jockey Club College of Veterinary Medicine and Life Sciences, City University of Hong Kong, Hong Kong, China; 6 School of Public Health, Peking University, Beijing, China

**Keywords:** Family health, Reliability, Validity, Psychometrics

## Abstract

**Background:**

With the release of the Health China Action (2019–2030), family health is receiving increasing attention from experts and scholars. But at present, there is no family health scale in China that involves multidimensional and interdisciplinary commonality.

**Aim:**

To translate a Short Form of the Family Health Scale (FHS-SF) and to test the reliability and validity of the Chinese version of the FHS-SF.

**Method:**

A Short Form of the Family Health Scale was Chinese translated with the consent of the original author. A total of 8912 residents were surveyed in 120 cities across China using a multistage sampling method, with gender, ethnicity, and education level as quota variables. Seven hundred fifty participants were selected to participate in this study, and 44 participants were randomly selected to be retested 1 month later.

**Results:**

The Cronbach’s alpha of the Chinese version of a Short Form the Family Health Scale was 0.83,the Cronbach’s alphas of the four subscales ranged from 0.70 to 0.90, the retest reliability of the scale was 0.75, the standardized factor loadings of the validation factor analysis were above 0.50, GFI = 0.98; NFI = 0.97; RFI = 0.95; RMSEA = 0.07, all within acceptable limits.

**Conclusion:**

The Chinese version of a Short Form the Family Health Scale has good reliability and validity and can be used to assess the level of family health of Chinese residents.

**Supplementary Information:**

The online version contains supplementary material available at 10.1186/s12875-022-01702-1.

## Introduction

With the release of the Health China Action (2019–2030), family health is receiving increasing attention from experts and scholars [1]. Family health is “a resource at the level of the family unit that develops from the intersection of the health of each family member, their interactions and capacities, as well as the family’s physical, social, emotional, economic, and medical resources.” [2]. A healthy family promotes a sense of belonging among family members [3] and fosters the ability of family members to care for each other and meet life’s responsibilities [4]. In addition, family communication can also improve the health-related quality of life among family members [5]. Families have an important role in maintaining health and preventing disease because family members may support each other at all stages of life in ways that other systems cannot [6]. In fact, the economic value of care provided by families over the lifetime of an individual is much greater than that of the health care system [7]. And with the major changes in China’s economic and social system since the 1980s, family health has faced serious challenges [8].

As family health research continues to evolve, the measurement of family health is receiving increasing attention [9]. Previous studies have not found self-assessment scales involving multidimensional and interdisciplinary commonality in family health. Simic-Ruzic et al. measured family health by semi-structured interviews in terms of dimensions such as emotional state, communication, boundaries, alliances, adaptability and stability, and family skills [10], however, the scale used in this study were other assessment scale, which was more cumbersome to operate and not conducive to the popularization of family health research. So more researchers tend to use self-assessment scales to measure family health, such as the Family Assessment Scale [11], the Family Adaptability and Cohesion Rating Scale [12], and the Family Functioning Scale [13], but these scales only measure one aspect of family health: family functioning. Further research led researchers to realize that family health should not be limited to only one dimension of family functioning, so Weiss-Laxer et al. studied family health in terms of individual family members’ health status, behavior, and health care utilization [2], while Novilla et al. considered family-level factors such as family structure, composition and income together [14]. Which were mainly caused by the non-existence of validated measures of family health.

Crandall et al. constructed a family health measurement network by inviting interdisciplinary family health experts using the Delphi method, and based on this resource network, they constructed a long-form of Family Health Scale containing four dimensions: family/social and emotional health processes, family healthy lifestyles, family health resources, and social support outside the family. The scale has high reliability and validity, however, due to the large number of scale items, fewer items can reduce subjects’ fatigue responses and increase the completion rate in practical studies [15]. Therefore, a short form of the Family Health Scale was further composed by taking the 2–3 items with the highest loadings from each of the four dimensions [16]. This short form is only available to adults who are 18 years of age or older. The result of the study indicates that a Short Form of the Family Health Scale has good reliability and validity.

At present, there is no family health scale in China that involves multidimensional and interdisciplinary commonality. Therefore, this study aims to translate the Family Health Scale-Short Form (FHS-SF) compiled by Crandall et al., and test its reliability and validity to form a Chinese version of the FHS-SF, so as to provide a quantitative tool for assessing family health problems in China.

## Methods

### Sample

The survey was conducted from May 2021 to September 2021, using a multi-stage sampling method that directly included the provincial capitals of 23 Chinese provinces and 5 autonomous regions, 4 municipalities directly under the central government (Beijing, Tianjin, Shanghai, Chongqing), and 2–6 cities in each of the non-capital prefecture-level administrative regions of each province and autonomous region using the random number table method, for a total of 120 cities. At least one surveyor or one survey team was recruited in each city, with each surveyor liable for collecting 30–90 questionnaires and each team responsible for collecting 100–200 questionnaires. The enumerators were required to obtain a sample with gender, age, and urban/rural distribution that generally matched the demographic characteristics based on the results of the “7th National Census, 2021.” The study was ethically reviewed (JNUKY-2021-018). The inclusion criteria were adults aged ≥18 years who all signed an informed consent form and voluntarily participated in this study. A total of 8912 residents were surveyed, and a total of 750 cases were sampled based on the data from the 7th National Census, and the sampling method studied by Crandall et al. using gender, nation, and education level as quota variables (see Table 1).

**Table 1:** Sampling frame

	Male (384)										Female (366)									
	Han (350)					Minority (34)					Han (333)					Minority (33)				
Education	1	2	3	4	5	1	2	3	4	5	1	2	3	4	5	1	2	3	4	5
n	9	94	131	57	59	1	9	13	5	6	10	89	124	54	56	1	9	12	5	6

Education: 1: illiterate; 2: primary; 3: junior high school; 4: high school; 5: university, *n* Number of people, *Han* Han nationality, *Minority* Minority nationality

### Measures

#### The general information questionnaire

The researcher prepared the general information questionnaire, which included the gender, age, nation, marital status, permanent residence, household registration, highest educational level, occupational status, number of siblings, and monthly per capita household income.

#### The Chinese version of FHS-SF

The Family Health Scale was developed by Crandall and Weiss-Laxer et al., and was designed to develop a tool that would effectively measure family health. The instrument consists of a Long Form of the Family Health Scale (FHS-LF) and a Short Form of the Family Health Scale (FHS-SF), which contains four dimensions: Family/social/emotional health processes, Family healthy lifestyle, Family health resources, and Family external social supports, while the Short-Form Scale consists of 2–3 items with higher factor loadings and weights drawn from each dimension. Among them, A1, A2, and A5 belong to the family/social/emotional health process dimension, A3 and A4 belong to the family healthy lifestyle dimension, A6, A9, and A10 belong to the family healthy resource dimension, and A7 and A8 belong to the family external social support. The FHS-SF uses a five-point Likert-type scale, with A6, A9 and A10 being reverse scored. Crandall et al.’s study found that the Cronbach’s alpha of the FHS-SF was 0.80. This study will carry out the Chineseization of the FHS-SF.

### Translation process

#### Translation and back-translation of the scale

Authorization for translation and use was obtained from the authors of the FHS-SF, and the scale was translated independently by 2 researchers (1 master in medicine and 1 master in translation). Afterward, 1 medical-oriented master and 2 translators participated together to compare and discuss the similarities and differences between the 2 translations to form the first draft of the Chinese version of the scale. Then 2 master’s degree students in translation were invited to back-translate the first draft of the Chinese version of the scale separately without knowing the specific contents of the scale. The researcher and the 2 back-translators were involved in formulating the back-translated version of the FHS-SF. All members involved in the translation and back-translation were then asked to discuss the similarities and differences between the original scale, the first draft of the translation, and the back-translated scale to ensure that they were equivalent and to avoid ambiguity.

#### Cultural debugging of the scale

Five experts in the field of family health were invited to form a panel of experts, who were asked to comment on and revise the formulation of the items in the light of the scale’s target audience and the cultural background of our country, and to propose amendments. The experts were asked to judge whether the content of each entry was in line with the current situation of families in China, to make adjustments to inappropriate points, and to make certain modifications based on our language expression habits without changing the original meaning, such as replacing “We help each other to change in a healthy direction” with “We help each other make healthy changes”, “My family did not have enough money at the end of the month after bills were paid” is changed to “After covering basic living expenses, our family has no spare money.” Finally, all 10 items are retained to form a Chinese version of the Family Health Scale.

Twenty-five people of different age groups were selected for the pre-survey in May 2021. The survey instruments were a paper version of the General Information Questionnaire and a Chinese version of the Family Health Short Form, and the subjects were asked about the clarity and comprehensibility of the entries after completing the survey.

### Statistical analysis

Data were analyzed using the SPSS 22.0 (IBM Corp., Armonk,NY, United States) and AMOS 22.0 (IBM Corp., Armonk,NY, United States). Data with questionnaire response lengths shorter than a quarter digit, inconsistent logic checks, incomplete information, duplicate fills, and data where the options checked were all the same or had a regularity were removed using SPSS 22.0. Descriptive statistics were used to describe the frequency and percentage of the sample on social-demographic characteristics. The correlation coefficient, extreme group method, and CITC method were used for item analysis. The correlation coefficient method required that correlations of items with coefficients *r* < 0.4 and *p* > .5 associated with the total scale score be dropped; the extreme group method required that items with t-values obtained using independent sample t-tests in the high (highest 27%) and low (lowest 27%) subgroups be dropped if the differences were not significant. The extreme group method requires that items with t-values obtained using independent sample t-tests in the high and low groups be removed if the difference is not significant. In addition, the CITC method requires that if the Cronbach’s alpha coefficient of an item increases significantly after deletion, the item will be less internally relevant and should be deleted [17].ANOVA was used to test for differences in family health scores at different levels of variance. All data were tested using a two-sided test, and *p* < 0.05 indicated that the differences were statistically significant.

Internal consistency Cronbach’s alpha coefficient, and retest reliability were used to test the reliability of the scale,and values ≥0.70 were considered to be good reliability [18]. Content validity was used to test the validity of the scale, on the one hand, the content validity of the original scale has been tested, and on the other hand, a panel of experts from different fields was formed to ensure the content validity of the scale [19]. In addition, confirmatory factor analysis was performed using AMOS 22.0 to test the structural validity of the scale. Fit indices included chi-squared over degrees of freedom (CMIN/DF) (values < 3 indicated a good fit [20], while some researchers consider values < 5 to be equally acceptable [17]), goodness-of-fit index (GFI) (values > 0.85 indicated an acceptable fit) [21], normed fit index (NFI) (values > 0.9 indicated a good fit) [22], relative fit index (RFI) (values > 0.9 indicated an acceptable fit) [23], root-mean-square error of approximation (RMSEA) (values < 0.08 indicated an acceptable fit) [21]. It was found that having a good fit such as CMIN/DF, RMSEA and GFI does not necessarily mean that the model is correct and reliable, that chi-square tests are better suited for small samples, and that chi-square tests lose their efficacy with large sample sizes, a time when other results including incremental fit indices, such as NFI and RFI, prove to be more informative indicators [24].

## Results

### Demographic characteristics of the sample

Among the 750 surveyed residents, 384 (51.2%) were male and 366 (48.8%) were female; 571 (76.1%) were married; 416 (55.5%) were urban residents and 334 (45.5%) were rural residents; 294 (39.2%) were non-agricultural households and 456 (60.8%) were agricultural households; 248 (33.0%) had high school education or above; 180 cases (24.0%) were aged 60 and above; 140 cases (18.7%) had a monthly per capita household income of RMB 1500 and below, and 46 cases (6.1%) had a per capita household income of RMB 10,501 and above; The two categories with the highest number of occupations were those with no fixed occupation and those in employment, while the two lowest categories were retirees and students (see Table 2).

**Table 2:** General demographic characteristics of the surveys

Variables	n	Percentage (%)	Variables	n	Percentage (%)
Gender			Age (years)		
Male	384	51.2	19–25	71	9.5
Female	366	48.8	26–30	62	8.3
Marital status			31–35	40	5.3
Unmarried	116	15.5	36–40	52	6.9
Married	571	76.1	41–45	129	17.2
Divorced	17	2.3	46–50	118	15.7
Widowed	46	6.1	51–55	75	10.0
Permanent residence			56–59 ≥60 Number of siblings	23	3.1
Urban	416	55.5		180	24.0
Rural	334	45.5			
Household registration			0	72	9.6
Non-agricultural	294	39.2	1	148	19.7
Agriculture	456	60.8	2	169	22.5
Educational level			≥3	361	48.1
Illiterate	21	2.8	Monthly per capita household income		
Primary school	201	26.8	≤1500	140	18.7
Junior high school	280	37.3	1501–3000	170	22.7
High school	121	16.1	3001–4500	164	21.9
University	127	16.9	4501–6000	121	16.1
Occupational status			6001–7500	55	7.3
Students	68	9.1	7501–9000	33	4.4
Employment	263	35.1	9001–10,500	21	2.8
Retirees	112	14.9	≥10,501	46	6.1
No fixed occupation	307	40.9			
Nation					
Han nationality	683	91.1			
Minority nationality	67	8.9			

*n* Number of people

### Response to each item of the Chinese version of the FHS-SF

The scores of each item in the Chinese version of the FHS-SF were mainly concentrated in 3 and 4. The total scores ranged from 10 to 50, with higher scores implying better family health status. The mean score of the total score of the Chinese FHS-SF was 38.47 ± 6.11 (Mean ± SD), indicating good family health status, as shown in Table 3.

**Table 3:** Response to each item of the Chinese version of the FHS-SF

Item	Score					Mean ± SD
	1(%)	2(%)	3(%)	4(%)	5(%)	
A1	2 (0.3)	29 (3.9)	142 (18.9)	327 (43.6)	250 (33.3)	4.06 ± 0.84
A2	3 (0.4)	38 (5.1)	167 (22.3)	282 (37.6)	260 (34.7)	4.01 ± 0.90
A3	3 (0.4)	23 (3.1)	137 (18.3)	300 (40.0)	287 (38.3)	4.13 ± 0.84
A4	3 (0.4)	31 (4.1)	156 (20.8)	301 (40.1)	259 (34.5)	4.04 ± 0.87
A5	2 (0.3)	23 (3.1)	143 (19.1)	330 (44.0)	252 (33.6)	4.08 ± 0.82
A6	25 (3.3)	100 (13.3)	171 (22.8)	187 (24.9)	267 (35.6)	3.76 ± 1.17
A7	12 (1.6)	26 (3.5)	199 (26.5)	370 (49.3)	143 (19.1)	3.81 ± 0.84
A8	18 (2.4)	37 (4.9)	166 (22.1)	324 (43.2)	205 (27.3)	3.88 ± 0.95
A9	57 (7.6)	182 (24.3)	218 (29.1)	171 (22.8)	122 (16.3)	3.16 ± 1.18
A10	35 (4.7)	113 (15.1)	204 (27.2)	207 (27.6)	191 (25.5)	3.54 ± 1.16
Total score						38.47 ± 6.11

### Item analysis

Pearson correlation was used to examine the correlation between the scores of each item and the total score. The results showed that there were significant and high correlations between the scores of the five items from the Family/social/emotional health processes dimension and the Family healthy lifestyle dimension of the FHS-LF and the total score, with correlation coefficients were 0.77–0.80 and 0.74–0.76, respectively, while the Family health resources and Family external social supports dimensions showed moderate significant correlations, with correlation coefficients of 0.48–0.57 and 0.57–0.59, respectively. Using the highest 27*%* and the lowest 27*%* of the total scale scores as the boundaries between the high and low subgroups, independent samples t-tests revealed significant differences (*p* < 0.001) between the scores of the high and low subgroups on each item for both scales. The Corrected item total correlation (CITC) were all above 0.30, and the combination of the deleted Cronbach’s alpha showed that the internal consistency coefficients did not change much after the deletion of the items [25] (see Table 4). The results of item analysis indicate that the Chinese version of a short form of the Family Health Scale (FHS-SF) has good discriminatory power.

**Table 4:** Corrected item total correlation of the surveys

Item	M	Total correlation	α
A1	34.41	0.71	0.80
A2	34.45	0.69	0.80
A3	34.34	0.69	0.80
A4	34.42	0.66	0.81
A5	34.39	0.74	0.80
A6	34.70	0.36	0.83
A7	34.66	0.49	0.82
A8	34.58	0.45	0.83
A9	35.31	0.31	0.84
A10	34.92	0.42	0.83

M = Mean value of scale after deletion; α =Cronbach’s alpha after deletion

### Reliability analysis of the Chinese version of the FHS-SF

The Cronbach alpha of the Chinese version of the FHS-SF was 0.83, the Cronbach alpha of the Family/social/emotional health processes subscale was 0.90, the Cronbach alpha of the Family Healthy Lifestyle subscale was 0.83, the Cronbach alpha of the Family Health Resources subscale was 0.72, and the Cronbach alpha of the Family external social supports subscale was 0.70. To test the stability of the scales, 44 participants were re-sampled for the retest reliability survey, and the results showed that the retest reliability of the Chinese version of the FHS-SF after 1 month was 0.75.

### Validity analysis of the Chinese version of the FHS-SF

#### Content validity

The expert consultation method was used to measure the content validity of the Chinese version of the FHS-SF. The questionnaire was designed after a scientific and comprehensive review of books and literature, and expert consultations and discussions were held on June 7, June 11, June 15, June 18, July 3, and July 8, 2021, before the questionnaire was formally used. Expert consultation and discussion followed the methodology used by Latter et al. Experts were able to give written qualitative comments on each of the FHS-SF items and dimensions that were applicable to each consultation [26]. The experts consulted were all senior and regionally representative, and their specialties ranged from social medicine, health statistics, health care management, behavioral epidemiology, psychology, Human medicine, clinical medicine, pharmacology, nursing, sociology, etc. Therefore, the content validity of this scale can be guaranteed.

#### Structural validity

**Fig. 1:**
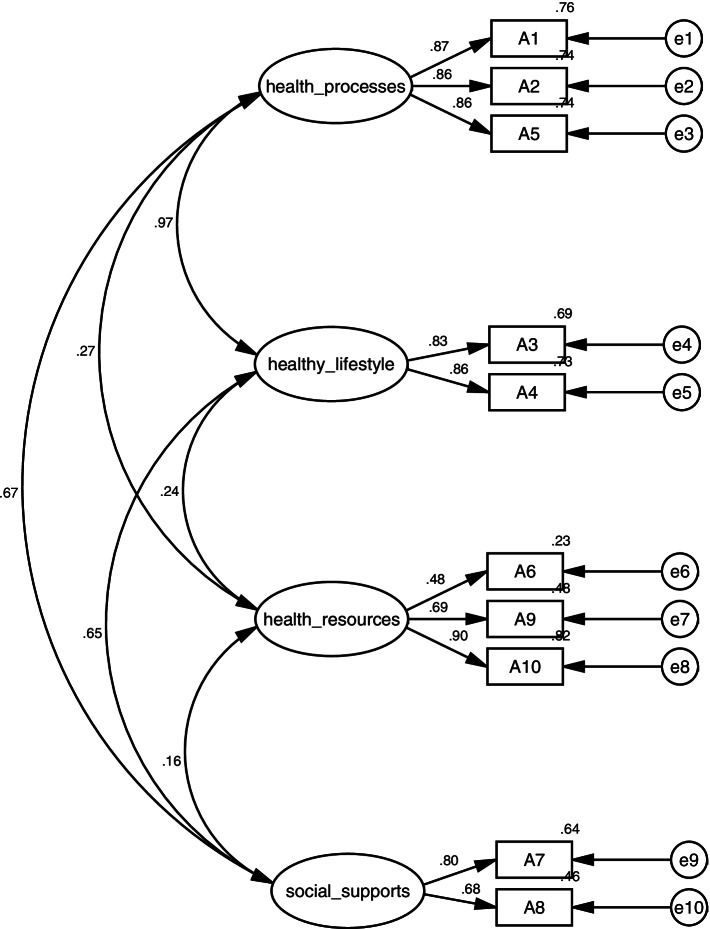
The validation factor analysis model for the Chinese version of the FHS-SF

The validation factor analysis was used to test the structural validity of the scale, and the scale was validated following the structural model of the original Family Health Short Form, and the results showed that the model fit indices were χ2/df = 4.28 < 5, GFI = 0.98 > 0.85,NFI = 0.97 > 0.90, RFI = 0.95 > 0.90, and RMSEA = 0.07 < 0.08, which is known from the fit indices that the model structural validity is good, and the results of the validation factor analysis are shown in Fig. 1.

### Differences in the total scores of FHS-SF at different levels of variables

Analysis of variance was used to test for differences in FHS-SF scores at each level of socio-demographic variables. Based on the study of Crandall et al., we selected gender, age group, marital status, and income level as subgroup variables, and found no significant differences in family health scores on gender, and income level. Family health scores were significantly lower in the age group younger than 40 years than in the age group older than 40 years. Significant differences were also found across marital status, specifically in the family health scores of the married group were significantly higher than those of the unmarried group (*p* < 0.001). See Table 5 for specific details.

**Table 5:** Differences in family health scores at different levels of variables

Variables	M ± SD	N	F	*P*
Gender				
Male	38.29 ± 0.31	384	0.66	0.42
Female	38.65 ± 0.32	366		
Age group				
Under 40	37.72 ± 6.15	225	4.81	0.03
Over 40	38.78 ± 6.07	525		
Marital status				
Unmarried	36.67 ± 6.41	116	5.20	0.01
Married	38.91 ± 6.05	571		
Divorced	36.18 ± 5.22	17		
Widowed	38.36 ± 5.56	46		
Income level				
Less than 3000	37.95 ± 5.77	310	2.04	0.11
3001–6000	38.51 ± 6.37	285		
6001–9000	39.32 ± 5.83	88		
More than 9000	39.55 ± 6.70	67		

## Discussion

### The Chinese version of the FHS-SF has good reliability and validity

The study of family health is of considerable importance as it promotes a sense of belonging among family members, fosters the ability of family members to care for each other and fulfill life responsibilities, and ultimately contributes to the overall development of society [27]. However, it has only received enough attention in China in recent years, and the definition of family health is confused, probably because of the lack of family health scales that involve multidimensional, interdisciplinary commonality. The purpose of this study is to translate the Family Health Scale-Short Form (FHS-SF) compiled by Crandall et al.. and test its reliability to form the Chinese version of the FHS-SF to provide a quantitative tool for assessing family health problems in China. When a scale is translated or to be used in another culture, it needs to be validated [28].

Item analysis showed that there was a significant difference between the scores of high and low subgroups on each item, indicating that the Chinese version of the FHS-SF has a good ability to discriminate between the high and low levels of the subjects’ family health. Meanwhile, there was a significant positive correlation between each item score and the total score, with correlation coefficients ranging from 0.48 to 0.80 (*p* < 0.001), all of which reached a significant level, and all items of the Chinese version of the FHS-SF met the measurement requirements [29], all of which were retained.

For the reliability test, the Chinese version of the FHS-SF reliability meets the measurement requirements. The Cronbach’s alpha of 0.7–0.8 is generally regarded as fairly good and 0.8–0.9 as very good [30]. It has been demonstrated that the Family Health Inventory has good reliability and validity. Cronbach’s α for this study ranged from .82 to .94, which is consistent with prior studies [31]. The Cronbach’s alpha of the Chinese version of the Family Health Scale was 0.83, and the Cronbach’s alpha of the subscales ranged from 0.70 to 0.90, indicating that the internal consistency of the scale was good. The retest reliability was 0.75, indicating good retest reliability and high stability of the measured family health scores. Compared to the English version of the FHS-SF, the Cronbach’s alpha and retest reliability of the Chinese version of the FHS-SF did not differ much, showing that the FHS-SF is a reliable instrument.

For validity testing, this study examined the validity of the Chinese version of the FHS-SF through structural validity and content validity. The modified model fit indices were: χ2/df = 4.28, GFI = 0.98, NFI = 0.97, RFI = 0.95, RMSEA = 0.07 < 0.08, which indicated that the model results had good validity. Previous studies have found that scales translated and validated in another society that have a good fit do not require any changes, which is consistent with the findings of the present study [32, 33]. The translated FHS-SF concepts of family health do not differ much from the original study and can be used in Chinese society.

Although entries were not removed in the study of the introduction of the Family Health Scale, not all scales translate so easily. For example, in a study of a cross-cultural nursing self-efficacy scale, Tian et al. found that a 5-point Likert-type scale was more appropriate than the initially used 10-point Likert-type scale while removing entries for some of the flawed attributes for better use in Chinese society [34]. In addition to this, in another study of translation and cultural accommodation for the Bayley-III Expressive Intercourse Scale, not only four items were deleted, but 12 items were revised and 12 items were added [35]. This phenomenon was also explained by Akram et al. in their study, who concluded that the differences between the translated scale and the original text depended on several factors, such as the concept of the study, the development of the original instrument method and accuracy, use of language, cultural differences, and other factors [36]. So, to further compare the cultural differences and accuracy of measurements between the Chinese version of the FHS-SF and the English version of the FHS-SF, we compared the findings of this study with those of previous studies done using the FHS-SF.

Previous studies have found cross-gender invariance in the FHS-SF, and in a study of heterosexual couples in the United States, no gender differences in family health scores were found [37], and the Chinese version of the FHS-SF also arrived at consistent findings, implying that there is little difference in perceived family health between men or women from the same family, and that it may still be the family as a system that plays an important role. Previous studies using the Family Health Scale have also revealed that household income levels in the United States are positively associated with family health, and that individuals with higher income levels have higher family health scores [38]. Socioeconomic status as a social determinant of health is strongly associated with family health [38], and although no differences in family health dimensions were found across income levels in this study, a trend toward progressively higher family health scores with higher income levels was also demonstrated, reflecting to some extent the role of economic level in promoting family health, with higher family income increasing resources and reduces other stressors [37], thus enhancing family health. Only this effect did not result in significant differences in the sampled population.

Significant differences were found for both age group and marital status, as evidenced by higher levels of family health among those older than 40 years of age and higher family health among those who were married than those who were unmarried. Crandall et al. also reported that age was not associated with FHS-SF scores [16]. However, a subsequent study presented the finding that the older the head of the household, the worse the FHS is [38], which was interpreted by Haehnel et al. to mean that older people have more responsibility and stress to bear and therefore have lower FHS. However, in Chinese society, we place a lot of emphasis on filial piety and we want the elderly to enjoy their old age, so the older people take on less family pressure and responsibility, and more responsibility and pressure is taken on by the younger and middle-aged people, thus showing a higher level of family health for people older than 40 years old. However, aging is associated with possible illness, death, or widowhood, all of which may affect family health [38], so future research is necessary to further explore the relationship between age and family health.

After comparing previous studies that used the FHS-SF with the data from this sample population, we found that the differences in family health did not change much at the level of different variables, where the age factor is likely to be related to different cultures, which reflects the good validity of the Chinese version of the FHS-SF.

### Shortcomings and outlook

There are still some shortcomings in this study: (i) the assessment tools for family health include not only the FHS, but also the Family Assessment Scale, the Family Adaptability and Cohesion Evaluation Scale, and the Family Functioning Scale, which should be included in future studies to facilitate cross-sectional comparisons among multiple scales and to conduct criterion validity studies [39]; (ii) the results of the chi-squared degrees of freedom ratio for the validation factor analysis failed to reach a higher standard (less than 3), so the scale’s structure needs further validation; (iii) This study did not compare a Long Form of the Family Health Scale with the Short Form, and future studies should further examine the structure and reliability of the Chinese version a Long Form of the Family Health Scale.

## Conclusions

The findings of this study provide a sufficient evidence that the Chine version of the FHS is valid and reliable to be used among the Chinese population. All items were retained and confirmed to be fit for the sample data.

## Supplementary Information

**Additional file 1.**

## Data Availability

The datasets generated and/or analysed during the current study are not publicly available due the data still needs to be used for other research but are available from the corresponding author on reasonable request.

## References

[1] Cheng XY (2020). Text analysis of health China action (2019-2030) based on policy tools. J Northeastern Univ (Social Science).

[2] Weiss-Laxer NS, Crandall A, Okano L, Riley AW (2020). Building a Foundation for Family Health Measurement in National Surveys: a modified Delphi expert process. Matern Child Health J.

[3] Kim W, Kreps GL, Shin CN (2015). The role of social support and social networks in health information-seeking behavior among Korean Americans: a qualitative study. Int J Equity Health..

[4] Hansen L, Rosenkranz SJ, Mularski RA, Leo MC (2016). Family perspectives on overall Care in the Intensive Care Unit. Nurs Res.

[5] Lim JW, Ashing-Giwa KT (2013). Is family functioning and communication associated with health-related quality of life for Chinese- and Korean-American breast cancer survivors?. Qual Life Res.

[6] Robinson LR, Holbrook JR, Bitsko RH, Hartwig SA, Kaminski JW, Ghandour RM (2017). Ifferences in health care, family, and community factors associated with mental, behavioral, and developmental disorders among children aged 2-8 years in rural and urban areas - United States, 2011-2012. D Morb Mortal Wkly Rep.

[7] Leiter V, Krauss MW, Anderson B, Wells N (2004). The consequences of caring - effects of mothering a child with special needs. J Fam Issues.

[8] Wan HW, Yu F, Kolanowski A (2008). Caring for aging Chinese: lessons learned from the United States. J Transcult Nurs.

[9] Fiese BH, Connell A, Doss B, Kaugars AS, Rhoades GK, Trentacosta CJ (2017). Introduction to the special issue: advances in methods and measurement in family psychology. J Fam Psychol.

[10] Simic-Ruzic B, Jovanovic AA (2008). Family characteristics of stuttering children. Srp Arh Celok Lek.

[11] Epstein N, Baldwin L, Bishop D (1983). The McMaster family assessment device. J Marital Fam Ther.

[12] Olson D (2011). FACES IV and the circumplex model: validation study. J Marital Fam Ther.

[13] Wang K, LI BY. (1998). Family health evaluation of the elderly in rural areas of Chengdu. Chin J Gerontol.

[14] Novilla M, Barnes M, Natalie G, Williams P, Rogers J (2006). Public health perspectives on the family: an ecological approach to promoting health in the family and community. Fam Commun Health.

[15] Tayama J, Ogawa S, Takeoka A, Kobayashi M, Shirabe S. Item response theory-based validation of a short form of the eating behavior scale for Japanese adults. Medicine. 2017;96(42):e8334. 10.1097/MD.0000000000008334 PMC5662414 29049248

[16] Crandall A, Weiss-Laxer NS, Broadbent E, Holmes EK, Magnusson BM, Okano L, et al. The family health scale: reliability and validity of a short- and long-form. Frontiers Public Health. 2020;8:587125. 10.3389/fpubh.2020.587125 PMC7717993 33330329

[17] Wu Y, Dong S, Li X, Xu H, Xie X (2022). The transcultural adaptation and validation of the Chinese version of the Duke anticoagulation satisfaction scale. Front Pharmacol.

[18] Webb NM, Shavelson RJ, Haertel EH, Rao CR, Sinharay S (2006). “4 Reliability Coefficients and Generalizability Theory,” in Handbook of Statistics.

[19] Chin YW, Lai PSM, Chia YC (2017). The validity and reliability of the English version of the diabetes distress scale for type 2 diabetes patients in Malaysia. Bmc. Fam Pract..

[20] Lt H, Bentler PM (1999). Cutoff criteria for fit indexes in covariance structure analysis: conventional criteria versus new alternatives. Struct Equ Model.

[21] McDonald RP, Ho MH (2002). Principles and practice in reporting structural equation analyses. Psychol Methods.

[22] Bentler PM, Bonett DG (1980). Significance tests and goodness of fit in the analysis of covariance structures. Psychol Bull.

[23] Ye ZJ, Qiu HZ, Li PF, Chen P, Liang MZ, Liu ML (2017). Validation and application of the Chinese version of the 10-item Connor-Davidson resilience scale (CD-RISC-10) among parents of children with cancer diagnosis. Eur J Oncol Nurs.

[24] Hammervold R, Olsson UH (2012). Testing structural equation models: the impact of error variances in the data generating process. Qual Quant.

[25] Iacobucci D, Neelamegham R, Hopkins N (1999). Measurement quality issues in dyadic models of relationships. Social Networks..

[26] Latter S, Maben J, Myall M, Young A (2007). Evaluating the clinical appropriateness of nurses' prescribing practice: method development and findings from an expert panel analysis. Qual Safe Health Care.

[27] Aslanturk H, Mavili A (2020). The sense of family belonging in university students from a single parent family compared with those from a two-biological-parent family. Curr Psychol.

[28] Chung JOK, Li WHC, Wei X, Cheung AT, Ho LLK, Chan GCF. Translation and psychometric evaluation of the Chinese version of the resilience scale for children with cancer. Health Qual Life Outcomes. 2021;19(1):232. 10.1186/s12955-021-01865-y PMC8487328 34600543

[29] Ml W. SPSS statistical application practice: questionnaire analysis and applied statistics: Science Press; 2003.

[30] Ml W (2010). Questionnaire statistical analysis practice: SPSS operation and application.

[31] Hanson CL, Crandall A, Barnes MD, Novilla ML (2021). Protection motivation during COVID-19: a cross-sectional study of family health, media, and economic influences. Health Educ Behav.

[32] Barati F, Sadegh-Moghadam L, Sajjadi M, Nazari S, Bahri N (2019). Validation of the Persian version of self-care tools for hypertension among older adults. Medicinski Glasnik.

[33] Leila SM, Mahshid F, Farahnaz MS, Fazlollah A, Moosa S, Akram F (2016). Validity and reliability of the Persian version of the brief aging perceptions questionnaire in Iranian older adults. Clin Interv Aging.

[34] Tian Y, Wang L, Xu Y, He Z (2021). The development of Chinese version of transcultural nursing self-efficiency scale: using Rasch model analysis. J Transcult Nurs.

[35] BangHeeJeong LSH (2009). 이유진. A preliminary study for the standardization of the Korean expressive communication scale of infant and toddler development, third edition. Korean J Dev Psychol.

[36] Kharazmi A, Brant JM, Sajjadi M, Moshki M, Moghadam LS (2020). Validation of the Persian version of family health climate scale (FHC-scale) in Iranian families. Bmc. Public Health..

[37] Crandall A, Barlow M (2022). Validation of the family health scale among heterosexual couples: a dyadic analysis. Bmc. Public Health..

[38] Haehnel Q, Whitehead C, Broadbent E, Hanson CL, Crandall A. What makes families healthy? Examining correlates of family health in a nationally representative sample of adults in the United States. J Fam Issues. 2021. 10.1177/0192513x211042841.

[39] de Beurs E, Carlier I, van Hemert A. Psychopathology and health-related quality of life as patient-reported treatment outcomes: evaluation of concordance between the brief symptom inventory (BSI) and the short Form-36 (SF-36) in psychiatric outpatients. Qual Life Res. 2021. 10.1007/s11136-021-03019-5. 10.1007/s11136-021-03019-5 PMC9023406 34729667

