# Supplementary material for: Reliability and validity of the Chinese version of a short form of the family health scale
Source: BMC Prim Care. 2022 May 6;23:108. doi: 10.1186/s12875-022-01702-1 (PMC9077878; doi:10.1186/s12875-022-01702-1)
Supplement: Supplementary file 1 — Additional file 1. [file 12875_2022_1702_MOESM1_ESM.docx]

**家庭健康量表**

**说明:请指出您在多大程度上同意或不同意以下陈述描述您的家庭。根据你认为的你的家庭来回答这些问题。**

**五点计分：非常不同意、有些不同意、既不同意也不反对、有些同意、非常同意**

|  | 非常不同意 | 有些不同意 | 既不同意也不反对 | 有些同意 | 非常同意 |
| --- | --- | --- | --- | --- | --- |
| A1在我的家庭里，我们互相支持 |  |  |  |  |  |
| A2在我的家庭里，我在家庭关系中感到有安全感 |  |  |  |  |  |
| A3在我的家庭里，在需要的时候，我们彼此帮助去寻求医疗服务(例如挂号) |  |  |  |  |  |
| A4在我的家庭里，我们互相帮助为了健康而做出改变 |  |  |  |  |  |
| A5在我的家庭里，在很艰难的时候，我们也保持希望 |  |  |  |  |  |
| A6在我的家庭里，我们不信任医护人员 |  |  |  |  |  |
| A7当我们在学校或工作中遇到问题时，我们家可以向家人以外的人寻求帮助 |  |  |  |  |  |
| A8如果我们需要经济上的帮助，我们家可以向家人以外的人借到钱(如：1000元) |  |  |  |  |  |
| A9在过去的12个月里，在负担基本的生活开销后，我们家就没有闲钱了 |  |  |  |  |  |
| A10在过去的12个月里，我家的住房不能满足家庭的需求 |  |  |  |  |  |

因素1:家庭社会和情感健康过程：4、11、18

因素2：家庭健康生活方式：16、17

因素3:家庭健康资源：23、31、32

因素4:家庭外部的社会支持：26、27

其中A6、A9、A10为反向计分，其他均正向计分，量表采用里克特式五点计分。
